# Effect of Fermentation on the Bioactive Compounds of the Black Soybean and Their Anti-Alzheimer’s Activity

**DOI:** 10.3389/fnut.2022.880361

**Published:** 2022-05-13

**Authors:** Umair Shabbir, Akanksha Tyagi, Hun Ju Ham, Fazle Elahi, Deog-Hwan Oh

**Affiliations:** ^1^Department of Food Science and Biotechnology, College of Agriculture and Life Sciences, Kangwon National University, Chuncheon, South Korea; ^2^Department of Biological Environment, College of Agriculture and Life Sciences, Kangwon National University, Chuncheon, South Korea

**Keywords:** anticholinesterase, neurodegeneration, metabolites, antioxidants, polyphenols

## Abstract

Black soybean is one of the nutritious crops and is being used in traditional medicines in Asian countries. In the present study, we fermented black soybean and screened against *in vitro* Alzheimer’s disease (AD) biomarkers such as cholinesterase enzymes, inflammatory factors, oxidative stress, and presence of γ-aminobutyric acid (GABA) levels. Firstly, we fermented black soybean with different lactic acid bacteria (LABs) and selected the *Pediococcus acidilactici* as the best LAB on the basis of GABA levels in the fermentate. We have found that black soybean fermented with *P. acidilactici* significantly inhibited the inflammatory factors (proteinase, protein denaturation, and lipoxygenase) and cholinesterase enzymes than non-fermented samples. An increase in the antioxidant capacity (FRAP, ABTS, and DPPH), anthocyanins, phenolics, flavonoids, and GABA content was also observed in fermented samples. Moreover, UHPLC-ESI-QTOF-MS/MS technique identified 38 bioactive components, including polyphenols, amino acids, and fatty acids. Among identified components, eight bioactive compounds were quantified, and an increase in the concentration of daidzein, genistein, glycitein, (+)-catechin, quercetin, and gallic acid was observed in fermented samples. However, the concentration of rutin and soyasaponin was higher in raw samples. These results indicated that fermentation of black soybean with *P. acidilactici* is a promising approach that can be used to develop functional foods to inhibit/prevent AD and other neurodegenerative diseases.

## Introduction

Oxidative stress is the oxidation of biomolecules caused by reactive oxygen species and can lead to cellular damage of proteins, DNA, and lipids. During oxidative stress conditions, more highly reactive species are produced that eventuate inflammation and aging (1). Both inflammation and oxidative stress are related and can activate glial cells (astrocyte and microglia), chemokines, and cytokines to damage the blood-brain barriers and lead to neurodegeneration (2, 3). Among neurological disorders, Alzheimer’s disease (AD) is the most common form of dementia that is considered to be caused by oxidative stress and neuroinflammation (3). According to Alzheimer’s Disease International, every 3 s, someone is developing AD or other types of dementia, and about 55 million people (globally) are suffering from these disorders. Low and middle-income countries suffer the most (around 60% cases), and this number is estimated to increase 71% by 2050. Progression in AD and dementia cases poses an inappropriate burden for society, family and the estimated annual worldwide cost is over US$ 1.3 trillion that forecasted to raise US$ 2.8 trillion by 2030 (4). Further, the formation of amyloid-beta (Aβ) peptides, neurofibrillary tangles, inflammatory signaling, oxidative stress in the nerve cells, alterations in neurogranin and neurotransmitters, including γ-aminobutyric acid (GABA) and acetylcholine (ACh), are the leading causes of AD (5). Loss in cholinergic neuro-transformation and neurons can decrease the ACh levels, and the cholinergic hypothesis states that the enhancement in the ACh and inhibition of acetylcholinesterase (AChE) and butyrylcholinesterase (BChE) levels can ameliorate the cognitive and memory functions in AD patients (6). Further, GABA (an inhibitory neurotransmitter) helps information processing nerve functioning and can moderate the dysfunctional Aβ effects in the hippocampus (7). Therefore, the elevation of antioxidants, ACh and GABA levels, and inhibition of inflammation, AChE, and BChE in the brain can be effective approaches to inhibit AD.

Black Soybean (*Glycine max* L.) is among the top-produced crops globally. It has therapeutic properties and is a rich source of anthocyanins, phenolic acids, and isoflavones that contributes to its anti-inflammatory, antioxidative, anticancer, and anti-diabetic properties. However, these beneficial activities not only depend on polyphenols but also the composition and concentration of individual compounds. Fermentation is a bio-conversion technique that can alter the ratio of nutritive and non-nutritive components of the black soybean. Moreover, triggers the levels of bioactive compounds such as phenolics, amino acids, fatty acids, and vitamins (8).

Identification and comparison of metabolites based on ultra-high performance liquid chromatography (UHPLC) coupled with mass spectrometry (MS) and the hybrid mass analyzer quadrupole-time-of-flight (QTOF) offer an excellent mass accuracy and measure the true fragmentation pattern of unknown metabolites. In the present study, we have examined the changes caused by the fermentation in the bioactive compounds of the black soybean using the UHPLC-ESI-QTOF-MS^2^. Furthermore, the effect of the fermentation on the bioactive compounds against Alzheimer’s disease (*in vitro* activities), including anticholinesterase, antioxidants, inhibition of inflammatory factors, and GABA levels, were also examined. According to our knowledge, this is the first study that implied a UHPLC-ESI-QTOF-MS^2^-based metabolite profiling approach to identify the whole metabolites profile of raw and fermented black soybean extracts. Additionally, raw and fermented black soybeans were also compared for the first time for their antioxidative and anticholinesterase, anti-inflammatory activity, and GABA levels. Therefore, the present study describes the promising potential of fermented black soybean to develop functional foods for the inhibition/prevention of AD and other neurological disorders.

## Materials and Methods

Black soybean (Se-Um variety) was obtained from the National Institute of Crop Science, Rural Development Administration-South Korea. Seeds of black soybean were pulverized into a fine powder (with electric mil), sieved, and stored at –20°C. *Pediococcus acidilactici* US1 and other LABs used for fermentation were collected from the Department of Food Science and Biotechnology, Kangwon National University-South Korea. The bacterial stock culture in MRS broth (Difco) containing 20% glycerol was stored at –80°C.

### Reagents and Chemicals

Analytical grade organic solvents (such as ethanol, methanol, etc.) were purchased from Daejung Chemicals & Metals Co., Ltd., Korea. Acetylcholinesterase (AChE) Electric eel (CAS 9000-81-1), acetylthiocholine iodide (ATCI), butyrylcholinesterase (BChE) equine serum lyophilized (CAS 9001-08-5), butyrylcholine iodide (BTCI), dithiobis nitrobenzoic acid (DTNB), galanthamine, 2,2’-azinobis-3-ethylbenzothiazoline-6-sulfonic acid (ABTS), trolox, daidzein, genistein, glycitein, (+)-catechin, rutin, quercetin, gallic acid, soyasaponin I, Folin–Ciocalteau’s reagent, cyanidine 3-o-glucoside chloride, Tris-HCl, lipoxygenase, aspirin, perchloric acid, linoleic acid, casein and 1,1-Diphenyl-2-picrylhydrazyl (DPPH) were obtained from Sigma.

### Sample Preparation

Black soybean powder (10 g/10mL each) and distilled water were mixed and autoclaved for 15 min at 121°C. The samples were cooled to 40°C and separated into different containers. About 5 mL of 2 × 10^7^ CFU/mL spores suspension of eight different bacterial strains [*P. acidilactici* US1, *Leuconostoc mesenteroides* US2, *Pediococcus pentosaceus* (FMC1), *Lactobacillus brevis* ATCC, *Enterococcus faecium* (EBD1), *Lactobacillus pentosus* (FMP1), *Lactobacillus plantarum* (FMR1), *Lactobacillus plantarum* (FMC2)] was collected from actively growing slants, inoculated into autoclaved samples. Afterward, inoculated samples were incubated at 37°C with 150 rpm (agitation) for 1–3 days. The media were centrifuged at 4,000 × *g* for 10 min and the supernatants were freeze-dried. The dried crude concentrated extracts were weighed to calculate the yield (%) and stored at –20°C until further use. The yield for the raw samples was 15.32%, and fermented samples was 18.87%. Initial screening for the best fermentation was done on the basis of antioxidant assays (DPPH, ABTS, and FRAP). Significant improvement was observed on the 2nd day of fermentation, and we have used it for further analyses.

### Sample Extraction

Extraction of samples (5 g each) was carried out with 70% ethanol (1:20 w/v) in an electric shaker at 50°C for 2 h. After that, extracted samples were centrifuged (at 4,000 × *g* for 15 min), the supernatants were collected, and the residues were re-extracted (up to 3rd extraction) following the same conditions. The supernatants were combined, filtered (0.20 μm), and evaporated at 50°C. Samples were stored at –20°C, and stock solutions were reconstituted in 10% DMSO or 70% ethanol for further use.

### γ-Aminobutyric Acid Detection

The GABA concentration of the ethanolic extract was determined using the method of Liu et al. (9) with slight modifications. The LC-20A HPLC equipped with a Hypersil ODS2 C18 column (4.6 mm × 250 mm, 5 μm) was used for GABA detection. The derivatization method was followed in which 1mg/mL sample extracts or 0.2 mL of GABA standard solution dissolved in distilled water (with different concentrations: 1, 0.5, 0.25, 0.12, 0.06 mg/mL) and mixed with 0.2 mL of dansyl chloride solution in a dark color volumetric flask and vortexed. Subsequently, samples or standards were derived for 1 h at 50°C in a water bath and cooled at room temperature. Then, samples were diluted with methanol (to a volume of 1.0 mL) and filtered with 0.45 μm membrane. Both samples and standards were adjusted at 8 ± 0.5 pH with 0.1 mol/L sodium bicarbonate. The standard curve of GABA was used to measure the content of GABA in samples, and data were presented in mg/g.

Conditions for HPLC were used as follows: an ODS2 Hypersil C_18_ (250 mm × 4.6 mm, 5 μm) chromatographic column; a-Q-grad pump; a flow rate of 1 mL/min; a column temperature of 30°C; methanol, mobile phase A; water, mobile phase B; and an injection volume of 2 μL.

### Anticholinesterase Assays

The AChE and BChE inhibitory activities of black soybean extracts were determined using the modified Ellman’s colorimetric method (10). A 96-well plate was used to conduct the reaction, and Galantamine (anticholinesterase drug) was used as a positive control. Acetylcholine iodide (ACTI) on Electric eel AChE and butyrylthiocholine chloride (BTCI) on horse serum BChE were employed as substrates of the reaction. Different concentrations (100–500 μg/mL) of sample solution dissolved in 10% DMSO was incubated with 20 μL solution of AChE (5.32 × 10^–3^ U) or BChE (6.85 × 10^–3^ U) in 150 μL of sodium phosphate buffer (100 mM; pH 8.0) for 45–50 min at room temperature. After that, 10 μL of 0.5 mM dithiobis nitrobenzoic acid was added, and ACTI (0.71 mM) or BTCI (0.2 mM) was mixed to start the reaction. The hydrolysis of these substrates was observed by the formation of yellow-colored 5-thio-2-nitrobenzoate anion at a wavelength of 412 nm using the spectrophotometer. The results were presented as IC_50_ (the sample concentration required for 50% inhibition) after calculation with the (below-mentioned) formula.

$$\%\,\mathrm{Inhibition}\,\mathrm{of}\,\mathrm{enzyme}=\frac{\text{absorbance}\,\mathrm{of}\,\mathrm{control}-\text{absorbance}\,\mathrm{of}\,\mathrm{sample}\times 100}{\text{absorbance}\,\mathrm{of}\,\mathrm{control}}$$

### Antioxidant Assays

#### Diphenyl-2-Picrylhydrazyl Assay

Diphenyl-2-picrylhydrazyl activity of ethanolic extract (1 mg/mL) was measured by following the method of Chen et al. (11) with slight modifications. Briefly, 100 μL of freshly prepared DPPH (500 μM) solution was mixed with 100 μL of sample extract, Trolox (standard), or blank (methanol) in a 24-well microplate. The reaction mixture was incubated for 30 min at room temperature, and absorbance was measured at 517 nm wavelength using the SpectraMax i3 plate reader (Molecular Devices Korea, LLC). The Trolox concentration plot with DPPH radical scavenging activity was used as a baseline curve. Results of DPPH were expressed as mg Trolox Equiv./g, DW using the below formula:

$$\mathrm{Radical}\mathrm{Scavenging}\mathrm{Activity}\left(\%\right)=\left({\text{A}}_{\text{c}}-A_{\text{e}}\right)/{\text{A}}_{\text{c}}\times 100$$

Where A_c_ is the absorbance value of blank (control), and A_e_ is the absorbance value of extract or Trolox.

#### Azinobis-3-Ethylbenzothiazoline-6-Sulfonic Acid Assay

Azinobis-3-ethylbenzothiazoline-6-sulfonic acid assay was determined by the method of Chen et al. (11) after slight changes. In short, 2.45 mmol/L of potassium persulfate was mixed with 7 mmol/L of ABTS solution (1:1, v/v) to prepare the ABTS stock solution and kept for 12–16 h in the dark (at room temperature). The ABTS^ +^ reagent was constantly diluted with methanol until 0.700 ± 0.010 absorbance at 734 nm wavelength. Then, 100 μL of sample extract (1 mg/mL), Trolox (standard), or blank (methanol) was mixed with ABTS^+^ solution (1 mL). The absorbance was measured at 734 nm wavelength using the SpectraMax i3 plate reader. The ABTS inhibition (%) was calculated using the same formula as for the DPPH assays, and values were presented as mg Trolox Equiv./g, DW.

#### Ferric Reducing Antioxidant Power Assay

The ferric reducing antioxidant power (FRAP)assay was determined by following the method of Zeng et al. (12) after slight alterations. Shortly, 0.1 mL of extracts (1 mg/mL) were combined with a FRAP reagent of 3.9 mL that was prepared using acetate buffer (50 mL, 0.3 M, pH 3.6), Tripyridyl Triazine (5 mL, TPTZ) solution (10 mM of TPTZ in 40 mM of HCl) and FeCl_3_ ⋅ 6H_2_O (5 mL, 20 mM)and incubated for 10 min at 37°C, the absorbance was measured at 593 nm. The FRAP results were expressed as mg Trolox Equiv./g, DW using the Trolox standard curve.

### Determination of Total Phenolic and Total Flavonoid Content

The determination of total phenolic (TPC) of ethanol extracts (1 mg/mL) was determined using the Folin–Ciocalteu method with the gallic acid standard described by Tyagi et al. (13) after slight modifications. Briefly, a 100-μL aliquot of each extract, standard or 95% methanol (blank), was mixed with 200 μL Folin-Ciocalteu reagent and incubated for 2 h. Afterward, 800 μL of Na_2_CO_3_ (700 mM) was added, and absorbance was measured at 765 nm using the SpectraMax i3 plate reader (Molecular Devices Korea, LLC). The values of TPC were expressed as mg gallic acid Equiv./(GAE)/g, DW.

Furthermore, total flavonoid content (TFC) was calculated by following the method of Tyagi et al. (13) with catechin as a standard. Shortly, 250 μL of ethanol extracts (1 mg/mL), standard or 95% methanol (blank) was mixed with NaNO_2_ (75 μL; 50 g/L) and 1 mL of distilled water. After the incubation for 5 min, AlCl_3_ (75 μL; 100 g/L) was mixed, and the mixture was settled for 6 min. Afterward, 600 μL of distilled water followed by 500 μL of 1 M NaOH was mixed, and absorbance was measured at 510 nm using the SpectraMax i3 plate reader. The values for TFC were expressed in mg catechin Equiv./g DW.

### Determination of Total Anthocyanin Content

The total anthocyanin content (TAC) was determined using the method of Lee et al. (14) with slight modifications. Briefly, 0.1 g of freeze-dried samples were dissolved in 10 mL of 60% ethanol containing 1% citric acid and vortexed. The absorbance was measured at 535 nm wavelength using the SpectraMax i3 plate reader. Cyanidine 3-*O*-Glucoside chloride (C3G) was used as a standard to create the standard curve, and TAC was measured as mg C3G Equiv./g DW.

### Inhibition of Inflammatory Factors (*in vitro*)

The inhibition of *in vitro* inflammatory factors (proteinase, protein denaturation, and lipoxygenase) by black soybean extracts was assessed using the methods of Gunathilake et al. (15) with slight modifications in the protocols. The freeze-dried (raw and fermented) samples were dissolved in 10% DMSO with different concentrations (25–500 μg/mL). Aspirin (a standard anti-inflammatory drug; 100 μg/mL) was used as a positive control, and 10% DMSO was used as a negative control.

#### Proteinase Inhibition

A reaction mixture containing 1mL of Tris HCl buffer (20 mM; pH 7.4) and 0.06 mg trypsin was pipetted with 1 mL of sample/standard and incubated at 37°C for 5 min. Then, 1 mL of 0.8% casein (w/v) was added and incubated for an additional 20 ± 2 min. Afterward, 70% of HClO_4_ (2mL) was added to stop the reaction, and the mixture was centrifuged for 10 min at 4,000 × *g* (4°C), and supernatants were collected. The absorbance was calculated at 210 nm wavelength, the inhibition percentage was calculated using the following formula, and data were presented as IC_50_.

%inhibition= 100×AcAs/Ac

Where Ac is the absorption value of negative control and As is the absorption value of the test sample.

#### Inhibition of Protein Denaturation

A reaction mixture consisting of 1 mL of sample or standard was added into the 1 mL of 1% aqueous solution of bovine albumin fraction and adjusted at 6.3 pH. Subsequently, incubated for 20 min at 37°C followed by heating for 5 min at 75°C and allowed to cool at room temperature. The absorbance was measured at 660 nm wavelength using the spectrophotometer. The inhibition (%) was calculated using the same formula for proteinase inhibition (mentioned above), and values were expressed as IC_50_.

#### Inhibition of Lipoxygenase

A mixture of 1 mL sodium borate buffer (0.1 M, pH 8.8) and 10 μL lipoxygenase (8,000 U/mL concentration) was incubated with 10 μL of sample or standard at room temperature for 5 min. About 10 μL of linoleic acid (10 mmol) substrate was added to the mixture to initiate the reaction and absorbance was measure at 234 nm. The inhibition (%) was calculated using the same formula for proteinase inhibition, and values were expressed as IC_50_.

### Identification of Bioactive Compounds

The bioactive compounds present in both raw and fermented samples were identified using the UHPLC-ESI-QTOF-MS^2^ (SCIEX Exion LC AD system, Framingham, MA, United States) following an earlier procedure used in our laboratory by Tyagi et al. (13). Both positive (ESI +) and negative (ESI-) ion modes were used to conduct mass spectrometry analysis. Accucore C18 column 100 mm × 3 mm (Thermo Fisher Scientific, Waltham, MA, United States) was used as an analytical column. In summary, UHPLC-ESI-QTOF-MS^2^ was attached with a controller, photoiodiode array detector, and autosampler components. Autosampler was used to inject the 10 μL sample (1 mg/mL) and eluted into the column with a binary mobile phase consisting of solvent A (water with 0.1% of formic acid) and solvent B (methanol). A flow rate (0.4 mL/min) with a linear gradient programmed for 25 min as demonstrated: 9–14% B (0–3.81 min), 14–15% B (3.81–4.85 min), 15% B (4.85–5.89 min), 15–17% B (5.89–8.32 min), 17–19% B (8.32–9.71 min), 19% B (9.71–10.40 min), 19–26% B (10.40–12.48 min), 26–28% B (12.48–13.17 min), 28–35% B (13.17–14.21 min), 35–40% B (14.21–15.95 min), 40–48% B (15.95–16.64 min), 48–53% B (16.64–18.37 min), 53–70% B (18.37–22.53 min), 70–9% B (22.53–22.88 min), and 9% B (22.88–25.00 min). Following the mentioned conditions, the scanning time was 1 s. The metabolomics workbench was used to identify the bioactive compounds in black soybean samples. Moreover, eight bioactive compounds were quantified against their standards (such as daidzein, genistein, glycitein, (+)-catechin, rutin, quercetin, gallic acid, and soyasaponin I) to check the alteration caused by fermentation in quantity.

### Statistical Analysis

Graphpad Prism 8.0 (GraphPad Software, San Diego, CA, United States) was used to analyze the data statistically. The difference in the mean values of black soybean extract activities was found using the one-way analysis (ANOVA) and Tukey’s test at *p* < 0.05 level of significance, and values were expressed as mean ± standard deviation (SD). The heat map and principal component analysis (PCA) were conducted using the ClusVis^1^ and Origin (data analysis software), respectively.

## Results

### Detection of γ-Aminobutyric Acid

**FIGURE 1:**
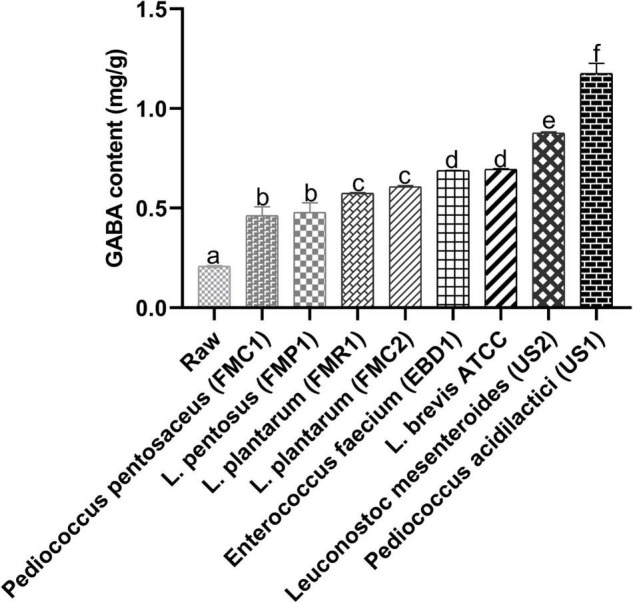
Screening of different LAB strains (48 h fermentation) based on gamma-aminobutyric acid (GABA). The sample concentration was 1 mg/mL; the values represent triplicate readings (mg/g) mean ± SD, and superscripts with different letters indicate a level of significance (*p* < 0.05).

γ-Aminobutyric acid is a naturally occurring amino acid and works as a principal inhibitory neurotransmitter. It maintains balance among neurotransmitter systems, and supplementation of GABA is considered a beneficial strategy to fight against AD (16). Therefore, we screened black soybean samples with different fermentation bacteria based on GABA concentration (Figure 1). Among eight different LAB strains, samples fermented with *P. acidilactici* US1 exhibited the highest GABA content (1.216 ± 0.06 mg/g). In contrast, GABA content in raw black soybean was 0.209 ± 0.08mg/g. Therefore, we selected *P. acidilactici* US1 among other LAB strains for further analysis based on these results.

### Anticholinesterase Activity

**FIGURE 2:**
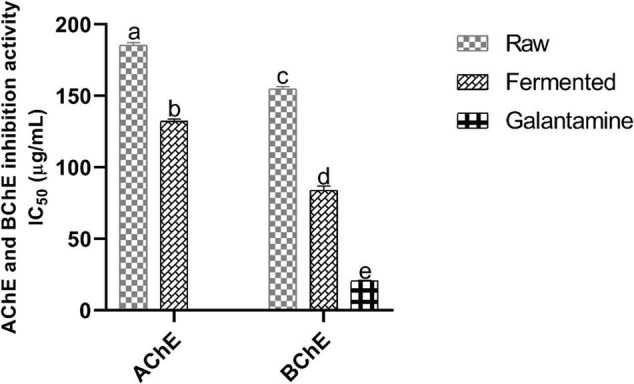
Anticholinesterase activity (*in vitro*) of black soybean samples. Values represent triplicate readings IC50 (μg/mL) and different superscripts denote significant differences (*p* < 0.05). AChE, acetylcholinesterase; BChE, butyrylcholinesterase.

The *in vitro* anticholinesterase (AChE and BChE) activity of sample extracts was examined, and results (IC_50_) were illustrated in Figure 2. A significant inhibition in AChE activity was observed in fermented samples (IC_50_ = 132.04 ± 1.45 μg/mL) than in raw (IC_50_ = 185.61 ± 1.47 μg/mL). Moreover, fermented samples also significantly inhibited BChE (IC_50_ = 84.58 ± 1.79 μg/mL) than raw (IC_50_ = 154.30 ± 1.83 μg/mL). Moreover, the effectiveness of the fermented samples was more significant against BChE than AChE. Therefore, fermentation of black soybean with *P. acidilactici* US1 can be used to increase the cholinergic neurotransmission *via* inhibition of AChE and BChE activities. However, Galantamine (IC_50_ = 20.82 ± 0.05 μg/mL) was inhibited more significantly than fermented samples.

### Antioxidant Activity (DPPH, ABTS, and FRAP), TAC, TFC, and TPC of Samples

The results of DPPH, ABTS, and FRAP with total phenolic, anthocyanin, and flavonoid contents of ethanol extracts were expressed in Table 1. Antioxidant assays were described in terms of mg Trolox equiv./100 g, DW, while TPC, TFC, and TAC in terms of gallic acid, catechin, and CG3 equivalent, respectively. In general, fermentation can increase the phenolic content and antioxidant capacity as studies suggest that phenolic components can scavenge the free radicals to suppress oxidative damage. We have observed that fermentation can effectively increase the black soybean’s health-related functionalities concerning antioxidant activities.

**TABLE 1:** Antioxidant activity (DPPH, ABTS, and FRAP), total flavonoid content (TFC), total phenolic content (TPC), and total anthocyanin content (TAC) of raw and fermented black soybean extracts.

Samples	DPPH (mg Trolox Equiv./g, DW)	ABTS (mg Trolox Equiv./g, DW)	FRAP (mg Trolox Equiv./g, DW)	TFC (mg Catechin Equiv./g, DW	TPC (mg Gallic Acid Equiv./g, DW)	TAC (mg CG3 Equiv./g, DW)
Raw BS	5.83 ± 0.75^a^	10.02 ± 1.63^a^	9.38 ± 1.47^a^	4.95 ± 0.76^a^	17.72 ± 0.28^a^	19.43 ± 0.27^a^
Fermented BS	9.93 ± 1.36^b^	16.93 ± 2.06^b^	15.83 ± 0.58^b^	9.03 ± 0.35^b^	22.96 ± 0.35^b^	21.84 ± 1.06^b^

*Values are presented as mean ± SD (n = 3), and different alphabets in each column represent the level of significance (p < 0.05). BS, black soybean; DW, dry weight.*

Significant increase in the DPPH (9.93 ± 1.36 mg Trolox Equiv./g, DW), ABTS (16.93 ± 2.06 mg Trolox Equiv./g, DW), and FRAP (15.83 ± 0.58 mg Trolox Equiv./g, DW) values were noticed for fermented samples as compared to raw. On the other hand, fermentation of black soybean significantly increased the TFC (9.03 ± 0.35 mg catechin Equiv./g, DW) than raw seeds (4.95 ± 0.76 mg catechin Equiv./g, DW). Furthermore, similar trends were observed for TAC (measured as mg CG3 Equiv./g, DW) and TPC (mg gallic acid Equiv./g, DW). About 3.41 mg/g in TAC and 5.24 mg/g in TPC were increased after the fermentation with *P. acidilactici* US1.

### Inhibition of Inflammatory Factors

#### Proteinase Inhibition

**FIGURE 3:**
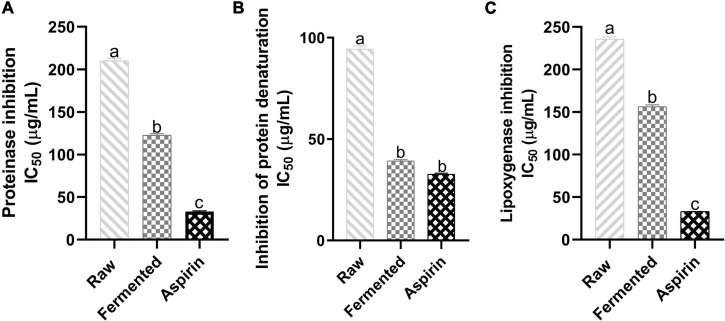
Effect of fermented and non-fermented black soybean samples against inflammatory factors. **(A)** Proteinase inhibition IC_50_ (μg/mL), **(B)** Inhibition of protein denaturation IC_50_ (μg/mL), **(C)** Lipoxygenase inhibition IC_50_ (μg/mL). The results represent triplicate values, and data marked with different letters are significantly different (*p* < 0.05).

Enzymes like proteinases contribute to inflammatory reactions, and the proteinases of leukocytes are involved in tissue damage development during the inflammation. Therefore, inhibition of proteinase can hinder inflammatory reactions. Figure 3A illustrates the antiproteinase activity (IC_50_) of black soybean samples. A significant enhancement in fermented and raw samples was observed where the fermented samples inhibited 122.31 ± 1.8 μg/mL and raw 209.52 ± 3.92 μg/mL of proteinase activity, and the values of aspirin (100 μg/mL) were 32.88 ± 0.75 μg/mL.

#### Inhibition of Protein Denaturation

Denaturation of protein tissues is recognized as inflammation markers because loss in the protein structure due to external stress, heat or presence of other compounds can lead to loss in their biological functionalities. Figure 3B shows the effect of black soybean samples against protein denaturation. Fermented samples showed significant protection against the bovine albumin denaturation (IC_50_ = 39.03 ± 1.79 μg/mL) than raw samples (IC_50_ = 94.91 ± 2.59 μg/mL). Values of fermented samples were comparable to aspirin (IC_50_ = 32.89 ± 0.05 μg/mL).

#### Inhibition of Lipoxygenase Activity

Lipoxygenase is one of the key enzymes in the biosynthesis of leukotriens and involved in inflammatory diseases. Figure 3C represents the anti-lipoxygenase activity (IC_50_) of sample extracts. Fermented samples showed a significant enhancement (156.29 ± 2.04 μg/mL) in the inhibition activity than non-fermented samples, and values for aspirin (100 μg/mL) were 32.99 ± 0.11 μg/mL.

### Identification of Metabolites

Total 38 bioactive compounds, including polyphenols, amino acids, and fatty acids, were tentatively identified in the fermented and non-fermented samples (Supplementary Table 1). Among tentatively identified compounds cyanidin 3-glucoside, cinnamic acid, galangin, genistin, and daidzin among polyphenols; L-valine, L-lysine, L-arginine, phenylalanine, γ-aminobutyric acid, and leucine among amino acids; α-linolenic acid, linoleic acid, lauric acid, palmitic acid, and arachidic acid among fatty acids; were predominately higher in fermented samples. Moreover, eight of the compounds were quantified (Table 2) and increased in the concentration of daidzein (0.3263 μg/mL), genistein (0.9014 μg/mL), glycitein (0.4091 μg/mL), (+)-catechin (1.1007 μg/mL), quercetin (0.0313 μg/mL), and gallic acid (0.1552 μg/mL) were observed in fermented samples. However, the concentration of rutin (0.0185 μg/mL) and soyasaponin (1.030.8 μg/mL) were higher in raw samples.

**TABLE 2:** Quantification of some of the bioactive compounds in non-fermented and fermented black soybean samples by UHPLC-ESI-QTOF-MS^2^.

S. no.	Sample	RT (min)	Concentration (μ g/mL)	Molecular formula	Component name
1	Raw BS	18.95	0.0117	C_15_H_10_O_4_	Daidzein
	Ferm BS	18.93	0.3263		
2	Raw BS	19.61	0.0381	C_15_H_10_O_5_	Genistein
	Ferm BS	19.59	0.9014		
3	Raw BS	19.83	0.0253	C_16_H_12_O_5_	Glycitein
	Ferm BS	19.94	0.4091		
4	Raw BS	10.23	0.0451	C_15_H_14_O_6_	(+)-Catechin
	Ferm BS	10.21	1.1007		
5	Raw BS	10.89	0.0185	C_27_H_30_O_16_	Rutin
	Ferm BS	10.88	0.0069		
6	Raw BS	19.24	0.0207	C_15_H_10_O_7_	Quercetin
	Ferm BS	19.22	0.0313		
7	Raw BS	3.07	0.0131	C_7_H_6_O_5_	Gallic acid
	Ferm BS	3.10	0.155.2		
8	Raw BS	22.02	1.0308	C_48_H_78_O_18_	Soyasaponin
	Ferm BS	22.19	0.6681		

*RT, retention time; BS, black soybean; Ferm, fermented.*

**FIGURE 4:**
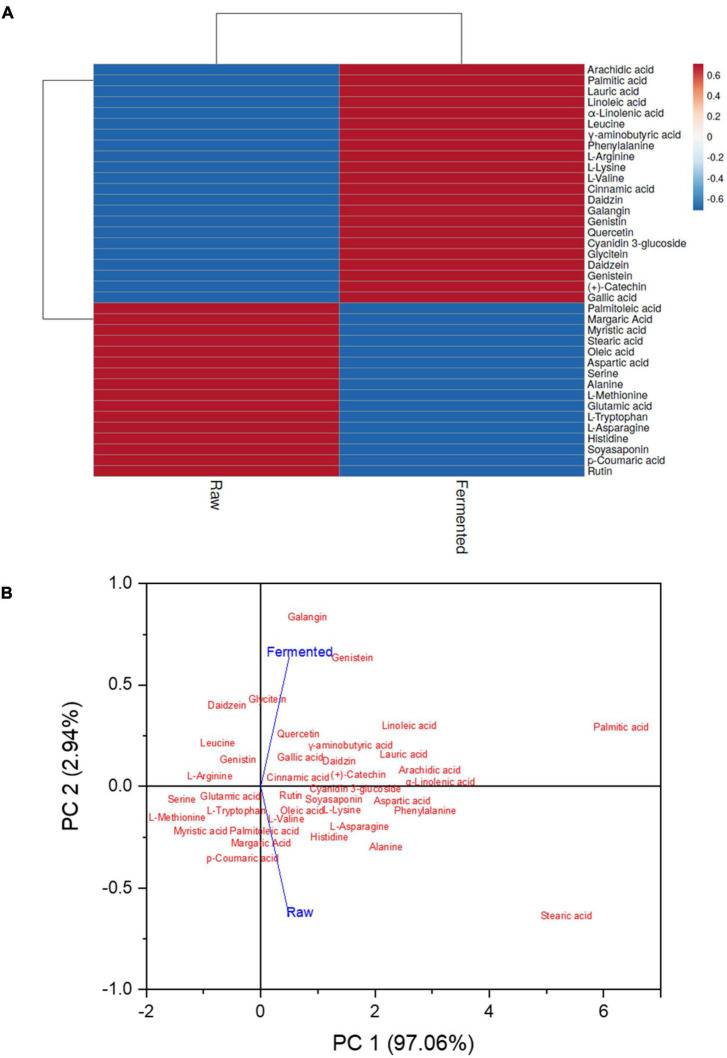
Identified bioactive compounds in the raw and fermented black soybean. **(A)** Heat map exhibits the varying amounts of identified compounds, from blue to red in samples, reflecting an increasing trend. **(B)** Principle component analysis (PCA) of samples was shown by comparing PC1 with PC2.

Heat map analysis was performed to show the alteration in the metabolites for clustering bioactive compounds based on their concentrations (Figure 4A). The color scheme ranged from blue to red, indicating concentration in increasing order. PCA was also applied for better interpretations and to avoid multicollinearity. Similar trends were noticed in the PCA analysis as interpreted in the heat map. It illustrated that the data from both of the groups were well split into distinct clusters. The two principal components (PCs) demonstrated 100% of the total data (PC1: 97.06%; PC2: 2.94%), which indicates that the prediction of the data was accurate (Figure 4B).

## Discussion

AD is generally characterized by memory or cognitive dysfunction due to the accumulation of Aβ peptides, neurofibrillary tangles, and a decrease in neurotransmitters such as GABA and acetylcholine followed by the loss of neurotransmission. Studies suggest that oxidative stress and neuro-inflammation are involved in the mechanism of Aβ-induced toxicity. Synthetic drugs used against AD (particularly for the inhibition of cholinesterase) have some adverse effects on the brain and can cause drowsiness, headache, and nausea. In the present study, we tested fermented black soybean (Se-Um variety) against the *in vitro* biomarkers of AD such as cholinesterase activity, inflammatory factors, oxidative stress, and GABA levels. Further, changes in raw to fermented black soybean metabolites were also investigated using the UHPLC-ESI-QTOF-MS^2^-based metabolite profiling approach.

Fermented samples exhibited higher GABA content that might be due to the stimulation in the glutamate decarboxylase reaction due to higher glutamic acid content. The GABA content mainly comes from the changes in the glutamic acid content during the fermentation (17). Moreover, Wu et al. (18) stated that lactic acid bacteria could also produce GABA during fermentation. Hwang et al. (17) observed a markedly increment in the GABA content and a decrease in glutamic acid after soybean fermentation. Similar results were noticed in this study (Figures 1, 4). A recent clinical study illustrated that supplementation of 200 mg GABA/day has improved or maintained cognitive function with verbal reasoning, working memory, and sustained attention of adult subjects aged 40 years (19). Therefore, fermentation with *P. acidilactici* US1 have potential for the enrichment of GABA in black soybean to treat neurological disorders.

Inhibiting cholinesterase activity is considered a promising approach for the treatment of AD (20). Both AChE and BChE enzymes catalyze the hydrolyses of cholinergic neurotransmitters, such as converting acetylcholine into choline and acetate. These hydrolyses disturb the neuronal functions and accelerate the accumulation of Aβ peptides to form the Aβ-AChE complex at the synaptic region of the hippocampus and lead to neuronal disorders. Moreover, BChE levels transform the benign plaques to malignant, resulting in neurons loss. Thus, an increase in the Ach levels and inhibition of the AChE and BChE can inhibit the formation of Aβ peptides. Szwajgier et al. (20) explained that polyphenols have an efficient ability to inhibit cholinesterase, and we have found that fermentation significantly enhanced the polyphenols level in black soybean. It is, therefore, the fermented samples have exhibited higher anticholinesterase activity. However, the inhibition mechanism of polyphenols for both enzymes can be different (21). Moreover, an increase in the concentration of TFC, TAC, TPC and antioxidant capacity (DPPH, FRAP, and ABTS) was observed after the fermentation of 48-hr, and these values were in accordance with Songlin et al. (8).

Inflammation is a complex process that leads to autoimmune, neurological, and other disorders. Natural anti-inflammatory agents are considered safer than drugs, and in the present study, we have tested the ability of black soybean to inhibit inflammatory factors (proteinase, lipoxygenase, and protein denaturation). Fermented samples exhibited higher inhibitory activity than raw samples, and it might be due to the presence of higher bioactive compounds (phenolic, flavonoid content, and antioxidants) (22). However, Eum and colleagues (23) documented that aglycones (e.g., daidzein, genistein, and glycitein) directly contribute to anti-inflammation, but bioactive factors, including TPC, TFC, and antioxidant activity, do not contribute directly. This study found higher bioactive factors: daidzein, genistein, glycitein, (+)-catechin, rutin, and p-coumaric acid (Table 2 and Figure 4) in fermented black soybean samples that exhibited higher inhibition of inflammatory factors.

We have quantified eight-different bioactive compounds and identified 38 untargeted metabolites (bioactive compounds), including amino acids, polyphenols, and fatty acids, and their peak area (equal to concentration). Fermented samples have exhibited higher concentrations of several bioactive compounds such as isoflavonoids (including daidzein, daizin, genistein, genistin, and glycitein), and these findings are in accordance with Li et al. (8), who observed an increment in the several bioactive compounds after the fermentation of whole soybean flour with *L. casei*. Polyphenols have antioxidative and anti-inflammatory properties and promote learning, cognitive and memory functions (24). Further, Suzuki et al. (25) documented that supplementation of leucine, phenylalanine, lysine, isoleucine, histidine, valine, and tryptophan to adults improved cognitive flexibility, psychosocial functioning, and attention. We have found that the black soybean (especially fermented with *P. acidilactici* US1) has higher amounts of essential and non-essential amino acids that can be used as a good source for the supplementation of amino acids to improve cognitive and psychological functions. We have identified 10 fatty acid and some of them can possess anti-neuro-inflammatory and neuroprotective activity, reduce amyloidosis, and promote neuronal maturation such as α-linolenic acid, linoleic acid (26), oleic acid (27), lauric acid (28), palmitic acid (29), and stearic acid (30). Thus, fermentation of black soybean can effectively inhibit cholinesterase enzymes, inflammatory factors, oxidative stress and can potentially enhance the GABA concentration.

## Conclusion

This study explored that black soybean has anticholinesterase, antioxidative and anti-inflammation properties. We also have found that fermentation with *P. acidilactici* US1 can be a potential bioconversion technique to enhance the metabolites of black soybean for the development of healthy functional foods to inhibit/prevent neurodegenerative diseases, including AD. However, the animal study is needed to support and substantiate the current findings.

## Data Availability Statement

The original contributions presented in the study are included in the article/Supplementary Material, further inquiries can be directed to the corresponding author.

## Author Contributions

US: writing—original draft, formal analysis, writing—review and editing, investigation, and conceptualization. AT: writing—review and editing and formal analysis. HH and FE: investigation. D-HO: supervision and conceptualization. All authors contributed to the article and approved the submitted version.

## Conflict of Interest

The authors declare that the research was conducted in the absence of any commercial or financial relationships that could be construed as a potential conflict of interest.

## Publisher’s Note

All claims expressed in this article are solely those of the authors and do not necessarily represent those of their affiliated organizations, or those of the publisher, the editors and the reviewers. Any product that may be evaluated in this article, or claim that may be made by its manufacturer, is not guaranteed or endorsed by the publisher.

## Footnotes

1
https://biit.cs.ut.ee/clustvis/
