# Supplementary material for: Effect of Fermentation on the Bioactive Compounds of the Black Soybean and Their Anti-Alzheimer’s Activity
Source: Front Nutr. 2022 May 13;9:880361. doi: 10.3389/fnut.2022.880361 (PMC9137038; doi:10.3389/fnut.2022.880361)
Supplement: Supplementary file 1 [file Table_1.DOCX]

Supplementary Table 1. Biochemical compounds identified by UHPLC−Q-TOF-MS^2^ from raw and fermented black soybeans

| **S.no.** | **Sample** | **RT (min)** | **Peak area** | **Precursor Mass** | **Found at Mass** | **Molecular formula** | **Identified component** |
| --- | --- | --- | --- | --- | --- | --- | --- |
| 1 | Raw | 10.23 | 2.34×10^4^ | 290.079 | 290.072 | [C_15_H_14_O_6_](https://pubchem.ncbi.nlm.nih.gov/#query=C15H14O6) | (+)-Catechin |
|  | Ferm | 10.21 | 5.72×10^5^ | 290.078 | 290.071 |  |  |
| 2 | Raw | 3.07 | 1.69×10^3^ | 170.022 | 169.014 | [C_7_H_6_O_5_](https://pubchem.ncbi.nlm.nih.gov/#query=C7H6O5) | Gallic acid |
|  | Ferm | 3.10 | 1.24×10^5^ | 170.022 | 169.012 |  |  |
| 3 | Raw | 8.09 | 1.19×10^3^ | 164.047 | 165.049 | [C_9_H_8_O_3_](https://pubchem.ncbi.nlm.nih.gov/#query=C9H8O3) | p-Coumaric acid |
|  | Ferm | Nd | Nd | Nd | Nd |  |  |
| 4 | Raw | 19.61 | 2.68×10^5^ | 270.241 | 270.052 | [C_15_H_10_O_5_](https://pubchem.ncbi.nlm.nih.gov/#query=C15H10O5) | Genistein |
|  | Ferm | 19.59 | 6.32×10^6^ | 270.241 | 270.052 |  |  |
| 5 | Raw | 18.95 | 6.18×10^4^ | 254.058 | 253.050 | [C_15_H_10_O_4_](https://pubchem.ncbi.nlm.nih.gov/#query=C15H10O4) | Daidzein |
|  | Ferm | 18.93 | 1.73×10^6^ | 254.058 | 253.050 |  |  |
| 6 | Raw | 19.83 | 5.09×10^5^ | 284.068 | 284.242 | C_16_H_12_O_5_ | Glycitein |
|  | Ferm | 19.94 | 4.61×10^6^ | 284.068 | 284.242 |  |  |
| 7 | Raw | 0.77 | 3.92×10^5^ | 484.077 | 484.078 | [C_21_H_21_ClO_11_](https://pubchem.ncbi.nlm.nih.gov/#query=C21H21ClO11) | Cyanidin 3-glucoside |
|  | Ferm | 0.78 | 5.43×10^5^ | 484.077 | 484.079 |  |  |
| 8 | Raw | 10.89 | 4.47×10^4^ | 610.153 | 610.50 | [C_27_H_30_O_16_](https://pubchem.ncbi.nlm.nih.gov/#query=C27H30O16) | Rutin |
|  | Ferm | 10.88 | 1.66×10^4^ | 610.153 | 610.50 |  |  |
| 9 | Raw | 3.82 | 9.10×10^3^ | 148.053 | 147.045 | C_9_H_8_O_2_ | Cinnamic acid |
|  | Ferm | 15.94 | 1.40×10^5^ | 148.053 | 147.045 |  |  |
| 10 | Raw | 19.24 | 4.57×10^4^ | 302.042 | 302.239 | C_15_H_10_O_7_ | Quercetin |
|  | Ferm | 19.22 | 6.90×10^4^ | 302.042 | 302.301 |  |  |
| 11 | Raw | 17.29 | 3.26×10^4^ | 432.106 | 431.098 | C_21_H_20_O_10_ | Genistin |
|  | Ferm | 17.29 | 7.60×10^4^ | 432.106 | 431.098 |  |  |
| 12 | Raw | 22.02 | 2.34×10^6^ | 942.518 | 943.102 | C_48_H_78_O_18_ | Soyasaponin |
|  | Ferm | 22.19 | 1.52×10^6^ | 942.518 | 943.102 |  |  |
| 13 | Raw | 18.28 | 1.60×10^6^ | 270.053 | 269.045 | C_15_H_10_O_5_ | Galangin |
|  | Ferm | 18.27 | 9.30×10^6^ | 270.053 | 269.045 |  |  |
| 14 | Raw | Nd | Nd | Nd | Nd | C_21_H_20_O_9_ | Daidzin |
|  | Ferm | 16.37 | 1.10×10^4^ | 416.111 | 415.103 |  |  |
| 15 | Raw | 1.29 | 1.30×10^4^ | 116.079 | 116.071 | [C_5_H_11_NO_2_](https://pubchem.ncbi.nlm.nih.gov/#query=C5H11NO2) | L-Valine |
|  | Ferm | 1.29 | 6.69×10^4^ | 116.079 | 116.071 |  |  |
| 16 | Raw | 0.89 | 7.60×10^3^ | 146.106 | 145.098 | [C_6_H_14_N_2_O_2_](https://pubchem.ncbi.nlm.nih.gov/#query=C6H14N2O2) | L-Lysine |
|  | Ferm | 0.87 | 4.10×10^4^ | 146.106 | 145.098 |  |  |
| 17 | Raw | 0.87 | 3.80×10^4^ | 174.112 | 173.104 | [C_6_H_14_N_4_O_2_](https://pubchem.ncbi.nlm.nih.gov/#query=C6H14N4O2) | L-Arginine |
|  | Ferm | 0.91 | 4.60×10^5^ | 174.111 | 173.103 |  |  |
| 18 | Raw | 0.91 | 1.29×10^5^ | 155.070 | 155.062 | C_6_H_9_N_3_O_2_ | Histidine |
|  | Ferm | 0.89 | 3.89×10^4^ | 155.070 | 155.062 |  |  |
| 19 | Raw | 10.93 | 2.54×10^4^ | 165.079 | 164.0717 | [C_9_H_11_NO_2_](https://pubchem.ncbi.nlm.nih.gov/#query=C9H11NO2) | Phenylalanine |
|  | Ferm | 10.91 | 8.60×10^4^ | 165.079 | 164.0717 |  |  |
| 20 | Raw | 0.98 | 5.70×10^4^ | 132.054 | 132.046 | C_4_H_8_N_2_O_3_ | L-Asparagine |
|  | Ferm | 0.97 | 1.28×10^4^ | 132.054 | 131.046 |  |  |
| 21 | Raw | 6.82 | 7.50×10^5^ | 204.090 | 203.082 | [C_11_H_12_N_2_O_2_](https://pubchem.ncbi.nlm.nih.gov/#query=C11H12N2O2) | L-Tryptophan |
|  | Ferm | 7.08 | 8.70×10^4^ | 204.090 | 203.082 |  |  |
| 22 | Raw | 1.02 | 5.87×10^5^ | 147.053 | 146.045 | C_5_H_9_NO_4_ | Glutamic acid |
|  | Ferm | 1.02 | 9.70×10^4^ | 147.053 | 146.045 |  |  |
| 23 | Raw | 1.03 | 2.50×10^4^ | 103.064 | 102.056 | C_4_H_9_NO_2_ | γ-aminobutyric acid |
|  | Ferm | 1.03 | 1.03×10^5^ | 103.064 | 102.056 |  |  |
| 24 | Raw | 2.13 | 5.70×10^4^ | 131.095 | 130.087 | C_6_H_13_NO_2_ | Leucine |
|  | Ferm | 2.14 | 1.20×10^5^ | 131.095 | 130.087 |  |  |
| 25 | Raw | 1.45 | 1.50×10^4^ | 149.051 | 148.043 | C_5_H_11_NO_2_S | L-Methionine |
|  | Ferm | 1.46 | 3.50×10^3^ | 149.051 | 148.043 |  |  |
| 26 | Raw | 1.01 | 1.30×10^4^ | 89.048 | 88.040 | C_3_H_7_NO_2_ | Alanine |
|  | Ferm | 1.03 | 1.26×10^3^ | 89.048 | 88.040 |  |  |
| 27 | Raw | 0.98 | 9.80×10^3^ | 105.043 | 104.035 | C_3_H_7_NO_3_ | Serine |
|  | Ferm | 1 | 1.63×10^3^ | 105.043 | 104.035 |  |  |
| 28 | Raw | 1.03 | 1.70×10^5^ | 133.038 | 132.030 | C_4_H_7_NO_4_ | Aspartic acid |
|  | Ferm | 1.01 | 7.50×10^4^ | 133.038 | 132.030 |  |  |
| 29 | Raw | Nd | Nd | Nd | Nd | [C_18_H_30_O_2_](https://pubchem.ncbi.nlm.nih.gov/#query=C18H30O2) | α-Linolenic acid |
|  | Ferm | 23.54 | 1.50×10^5^ | 278.225 | 277.217 |  |  |
| 30 | Raw | 21.83 | 3.90×10^6^ | 282.256 | 281.248 | [C_18_H_34_O_2_](https://pubchem.ncbi.nlm.nih.gov/#query=C18H34O2) | Oleic acid |
|  | Ferm | 22.31 | 3.60×10^6^ | 282.256 | 282.098 |  |  |
| 31 | Raw | 23.14 | 2.00×10^6^ | 280.241 | 279.233 | [C_18_H_32_O_2_](https://pubchem.ncbi.nlm.nih.gov/#query=C18H32O2) | Linoleic acid |
|  | Ferm | 23.15 | 5.30×10^6^ | 280.241 | 279.233 |  |  |
| 32 | Raw | 23.72 | 3.00×10^5^ | 200.178 | 199.17 | C_12_H_24_O_2_ | Lauric acid |
|  | Ferm | 23.71 | 3.20×10^5^ | 200.178 | 199.17 |  |  |
| 33 | Raw | 23.17 | 2.50×10^7^ | 256.240 | 255.232 | C_16_H_32_O_2_ | Palmitic acid |
|  | Ferm | 23.09 | 3.00×10^7^ | 256.240 | 255.233 |  |  |
| 34 | Raw | 22.32 | 2.50×10^7^ | 284.271 | 283.264 | C_18_H_36_O_2_ | Stearic acid |
|  | Ferm | 22.36 | 2.20×10^7^ | 284.271 | 283.264 |  |  |
| 35 | Raw | 23.63 | 1.20×10^6^ | 228.209 | 227.201 | C_14_H_28_O_2_ | Myristic acid |
|  | Ferm | 23.66 | 6.20×10^5^ | 228.209 | 227.165 |  |  |
| 36 | Raw | 22.81 | 2.10×10^6^ | 270.256 | 269.241 | C_17_H_34_O_2_ | Margaric Acid |
|  | Ferm | 22.91 | 2.30×10^5^ | 270.256 | 269.248 |  |  |
| 37 | Raw | 23.66 | 6.10×10^4^ | 312.307 | 311.295 | C_20_H_40_O_2_ | Arachidic acid |
|  | Ferm | 23.67 | 3.50×10^5^ | 312.307 | 311.296 |  |  |
| 38 | Raw | 22.18 | 1.80×10^6^ | 254.225 | 253.217 | C_16_H_30_O_2_ | Palmitoleic acid |
|  | Ferm | 22.23 | 4.30×10^5^ | 254.225 | 253.218 |  |  |

Ferm: fermented sample, RT: retention time
